# Adding L-Carnitine and Selenium to Methimazole in Graves’ Disease: A Prospective Randomized Trial on Thyroid Markers and Quality of Life

**DOI:** 10.3390/nu17162693

**Published:** 2025-08-20

**Authors:** Mattia Rossi, Letizia Meomartino, Marco Zavattaro, Gloria Selvatico, Ruth Rossetto Giaccherino, Loredana Pagano

**Affiliations:** 1 Endocrinology, Diabetology and Metabolism, Department of Medical Sciences, University of Turin, 10128 Turin, Italy; 2 Endocrinology, Diabetology and Metabolism, AO “Ordine Mauriziano di Torino”, 10128 Turin, Italy; 3 Endocrinology, Department of Translational Medicine, University of Piemonte Orientale, 13100 Novara, Italy

**Keywords:** Graves’ disease, selenium, carnitine, methimazole, hyperthyroidism

## Abstract

***Background***: The therapeutic response in Graves’ Disease (GD) remains largely unpredictable. Patients often experience persistent symptoms that are poorly correlated with thyroid hormone levels, an undefined treatment duration, and the need for long-term or definitive therapies. Based on the nuclear antagonistic properties of L-carnitine (LCT) on thyroid hormone action and the immunomodulatory role of selenium (Se), we aimed to assess the impact of adding a combined LCT and Se supplement to standard methimazole (MMI) therapy on the biochemical profile and quality of life (QoL) of patients with overt GD. ***Methods***: This multicenter prospective randomized trial enrolled 60 consecutive patients with newly diagnosed overt GD. Participants were randomized to receive either standard treatment with MMI alone (Control Group) or MMI plus the combined LCT/Se supplement (Intervention Group). TSH, fT3, fT4, and TSH–receptor antibodies (TRAb) levels were evaluated every two months for up to 24 months or until spontaneous remission or definitive therapy. At each visit, patients completed a symptom questionnaire addressing the frequency of typical thyrotoxic symptoms. ***Results***: No significant differences were observed between groups in the trend or time-to-normalization of TSH, fT3, and fT4 levels. However, the *Intervention Group* reached TRAb negativity significantly earlier (HR = 2.35 (1.14–4.81), *p* = 0.016), with a synergistic interaction with MMI therapy. MMI requirements were consistently lower in the *Intervention Group*, both in average dosage (*p* = 0.013) and cumulative dose (*p* = 0.020). The rate of spontaneous remission was significantly higher (OR = 11.22 (3.35–46.11), *p* < 0.001). Overall symptom burden did not differ significantly between groups; however, the supplement exerted an independent effect in reducing the severity of tremor, irritability, mood lability, heat intolerance, and exertional dyspnea. ***Conclusions***: Our findings suggest the clinical benefits of adding combined LCT and Se supplementation to MMI in the treatment of overt GD, including shorter disease duration, lower cumulative MMI exposure and earlier TRAb normality, that could positively influence TRAb-related prognostic outcomes.

## 1. Introduction

Graves’ disease (GD) is the most common cause of hyperthyroidism in iodine-sufficient areas, with a global prevalence of 1–1.5% [1].

This condition presents a heterogeneous clinical picture, and the severity of symptoms is only moderately correlated with thyroid hormone levels [2]. The clinical impact is significant both in terms of morbidity and mortality, particularly cardiovascular, with an excess risk of up to 49% [3], and in terms of the inevitably impaired quality of life of these patients [4,5].

GD is also known for its potential for spontaneous remission during treatment, with rates reaching 40–60% after 12–18 months of therapy [6]. However, the risk of relapse remains substantial, occurring in 50–70% of cases depending on the study [7]. Current guidelines recommend medical therapy with methimazole (MMI) as the first-line treatment [1]; however, the benefits of prolonged medical therapy beyond two years remain controversial [8,9]. Therefore, after two years of treatment or in the event of a relapse, definitive approaches (thyroidectomy or radioiodine therapy) are recommended, yet both have limitations.

The natural enantiomer of carnitine, L-carnitine (LCT), plays a crucial role in cellular energy metabolism [10]. While its supplementation is primarily of interest to athletes [11], basic and clinical studies dating back to the 1950s and 1960s first raised interest in its potential effects in the field of thyroidology [12,13,14,15]. In humans, LCT supplementation has been associated with improvements in hyperthyroid symptoms and peripheral biomarkers without reducing thyroid hormone levels or altering thyroidal radioiodine uptake. These findings support the hypothesis of a peripheral antagonistic effect rather than direct inhibition of thyroid function or hypothalamic–pituitary feedback. This hypothesis aligns with in vitro studies demonstrating that LCT inhibits both cellular and, to a greater extent, nuclear thyroid hormone entry [16]. Clinically, this knowledge has facilitated successful treatment of severe GD cases, including thyroid storm, with LCT [17,18,19].

GD represents a hypermetabolic state that results in saturation of physiological antioxidant systems and uncontrolled production of reactive oxygen species (ROS) in both peripheral tissues and the thyroid gland [20,21,22,23].

Selenium (Se) is a micronutrient incorporated as selenocysteine into selenoproteins, including thioredoxin reductases, glutathione peroxidases, and iodothyronine deiodinases [24,25,26,27]. In addition to its well-known role in thyroid hormone metabolism, selenoproteins exert immunomodulatory and antioxidant functions, particularly within the thyroid, where they are highly expressed [28].

Several studies have suggested that selenium supplementation facilitates more rapid restoration of euthyroidism, potentially by reducing oxidative stress exposure [29,30,31]. However, these findings are not universally consistent [24,32], possibly due to variations in baseline selenium status. Systematic reviews and meta-analyses indicate that selenium supplementation significantly reduces anti-thyroid peroxidase antibodies (TPOAb) in chronic autoimmune thyroiditis [33]. Similar but less conclusive results have been reported regarding its effects on TSH receptor antibodies (TRAb) levels [34,35,36]. The most widely accepted clinical application of selenium is in the treatment of mild Graves’ orbitopathy (GO) [37].

A recent uncontrolled pilot study on a small cohort of patients with subclinical GD receiving one month of LCT and selenium supplementation reported symptomatic improvement and reductions in anti-thyroglobulin (TgAb) and TPOAb levels without affecting thyroid hormone profiles. Notably, 60% of patients experienced symptoms and functional index worsening upon treatment discontinuation [38].

Our study aims to evaluate, in a randomized controlled setting, the effects of adding a combined LCT and Se supplement to standard MMI therapy in patients with overt GD. Specifically, we intend to assess the impact on thyroid hormone profiles and TRAb levels, as well as on patient-reported symptoms, thus evaluating its effect on quality of life.

## 2. Methods

### 2.1. Study Design

In this prospective, randomized, parallel group study we consecutively enrolled all patients who attended the outpatient clinics of the Endocrinology departments of the University Hospitals “Città della Salute e della Scienza di Torino” and “Ospedale Maggiore della Carità di Novara”, between 1 June 2021 and 30 June 2023 and met the following inclusion and exclusion criteria. A total of 60 participants were enrolled.

Inclusion Criteria:

- First diagnosis of overt GD (TSH < lower limit of normal range (LLN) for the reference laboratory, fT4 > upper limit of normal range (ULN) and/or fT3 > ULN, TRAb titer > ULN) with indication for initiating medical therapy with MMI.

Exclusion Criteria:

- Pregnancy;
- Age below 18 years;
- GO at enrollment;
- Reported intolerance and/or hypersensitivity reaction to any component of the studied supplement;
- Patients already requiring definitive therapy at enrollment;
- History of cardiovascular disease,
- History of anxiety and/or mood disorders.

### 2.2. Interventions

Enrolled patients were randomized into two treatment groups:

- Control Group (C) (30 patients): treated according to standard practice with MMI and beta-blockers only;
- Intervention Group (I) (30 patients): treated with MMI, beta-blockers, and a combined supplement containing selenium (83 mcg) and L-carnitine (500 mg).

Enrolled patients underwent clinical and biochemical evaluations every 2 months for up to 24 months. At each visit, thyroid function was assessed to adjust MMI dosage, and patients’ symptoms were evaluated using a specific questionnaire.

Early withdrawal from the study was foreseen in the following cases:

- Spontaneous remission: defined as normalization of TSH, fT4, and fT3 levels with concurrent TRAb negativity, confirmed after 2 months off therapy;
- Indication for definitive therapy: when the investigator determined that the patient required thyroidectomy or radioiodine therapy;
- Intolerance to treatments: side effects and/or intolerance were evaluated during each evaluation, allowing patients to withdraw from the study at any time and pursue alternative treatments. If intolerance to the supplement occurred within the first 2 months, the supplement was discontinued, and the patient was subsequently analyzed as part of the Control Group, following an as-treated approach.

#### More About Treatments

- *MMI*: MMI was administered in its standard 5 mg tablet formulation. According to international guidelines, an initial dose of 10–30 mg was prescribed, with titration during follow-up to reach euthyroidism, based on fT3 and fT4 levels, aiming for a maintenance dose of typically 5–10 mg. Patients obtained MMI from their local pharmacies. In Italy, only one formulation of MMI is commercially available (Tapazole © tablets), eliminating any source of bias.
- *Supplement*: The supplement (Tiroxil 0.4 ©) contained 83 µg of elemental selenium in the form of L-selenomethionine, together with 500 mg of L-carnitine, supplied as tablets with a recommended dosage of one tablet daily, irrespective of meals. It is commercially available in Italy and was supplied at no cost by the manufacturing company, Lo.Li. Pharma™ (Rome, Italy). The tablet was self-administered daily at home by the patients; therefore, no adherence monitoring strategies could be implemented. Yet, to enhance adherence, the supplement was periodically provided to patients. Reported side effects were limited to gastrointestinal issues such as diarrhea and nausea.

**Note:** To date, no evidence has arisen concerning risks associated with LCT or Se during pregnancy and/or lactation. Therefore, the supplement is not contraindicated in these situations. Consequently, women of childbearing age were not excluded from the trial, nor was contraception recommended. However, pregnant patients were excluded due to different therapeutic targets, preferential indication for propylthiouracil therapy, potential interference of pregnancy-related immune tolerance on antibody titer, and possible confounding effect on certain symptoms shared between the two conditions (e.g., palpitations, insomnia, emotional lability). For the same reasons, female participants were advised to promptly inform the investigators in case of a suspected pregnancy, to be withdrawn from the study and to assess treatment continuation on a case-by-case basis.

### 2.3. Biochemical Evaluations

At enrollment and every 2 months, serum levels of TSH, fT3, fT4, and TRAb were measured. TSH, fT3, and fT4 levels were determined using a chemiluminescent microparticle immunoassay (CMIA) on the “Alinity i” platform (Abbott Ireland, Diagnostics Division, Lisnamuck, Longford, Co. Longford, Ireland). Our laboratory reference ranges were 0.35 to 4.94 mUI/L for TSH, 1.58 to 3.9 ng/L for fT3, and 7.0 to 14.8 ng/L for fT4. TRAb levels were determined using an electrochemiluminescence immunoassay (ECLIA) with the “Elecsys Anti–TSHR” kit on the “Cobas E810” platform (Roche Diagnostics GmbH, Mannheim, Germany), based on a competitive principle. For TRAb, the manufacturer provides only an upper reference limit of 3.5 UI/L.

#### Methimazole Cumulative Dose

To assess cumulative exposure to methimazole (MMI), a variable termed “cumulative MMI dose” was defined and expressed in mg × months. This metric was calculated as the sum of the monthly MMI daily dosages for each patient across the follow-up period, reflecting the total burden of drug exposure over time. The calculation follows the same conceptual approach as pack-years used in quantifying cumulative exposure to tobacco smoking.

### 2.4. Symptoms and Quality of Life Assessment

At enrollment and every 2 months, a questionnaire assessing patient symptoms was administered. The questionnaire included 10 items: irritability, tremors, anxiety, palpitations, mood lability, insomnia, impaired concentration, exertion dyspnea, excessive sweating, and heat intolerance. Each symptom was scored on a 4-point scale (0 = absent, 1 = infrequent, 2 = frequent, 3 = constant), yielding a total Symptom Score (SS) ranging from 0 to 30, reflecting overall symptom burden and impact on QoL. This questionnaire has not undergone validation process was specifically designed for the study, based on the most common and debilitating symptoms of hyperthyroidism, which often drive the clinician to use additional drugs. The same tool was employed in the study that primarily inspired our protocol [38], and its use facilitated a direct and meaningful comparison with the available literature. We, then, assessed its internal consistency using Cronbach’s alpha at three main follow-up times (baseline, 6 months, and 12 months) in order to ensure the reliability of the instrument, to verify the coherence of responses across items and to support the interpretability of the derived scores within our study population.

Tables S13–S15 in Supplementary Materials summarize the results of this analysis, which, in our population, demonstrated a precise and reliable, yet not redundant, estimate for each follow-up time.

### 2.5. Clinical and Anamnestic Data Collection

At enrollment, the following data were recorded: age, sex, weight, height, body mass index (BMI), and smoking habits. At each 2-month visit, weight, BMI, presence of GO, MMI dosage, and the occurrence of treatment-related complications were recorded.

### 2.6. Endpoints and Outcome Assessment

This study aimed to explore the following primary endpoints:

1. Association between the treatment and rates of spontaneous resolution;
2. Higher cumulative incidence of TRAb negativity (TRAb < ULN);
3. Lower MMI cumulative dose;
4. Lower patients reported symptoms over time (lower AUC of overall Symptom Score).

As secondary endpoints, we also evaluated the following:

1. Variations over time of thyroid function markers and TRAb titer;
2. Cumulative incidence of the normalization of thyroid function indices (TSH > LLN, fT4 < ULN, fT3 < ULN);
3. Reduction of patient-reported symptoms over time, as single items.

### 2.7. Randomization and Blinding

Patients were assigned to each treatment group using a simple randomization process. The study was conducted as an open-label trial. A placebo tablet was not included in the control group; therefore, blinding techniques could not be implemented.

### 2.8. Statistical Analysis

Statistical analyses were performed using R software (version 4.2.2; R Core Team, 2022). Mean values and 95% confidence intervals were chosen as measures of central tendency for continuous variables. Categorical variables were described as absolute counts and percentages. The normal distribution of the variables was assessed with the Shapiro–Wilk test. At baseline, categorical variables were compared using the chi-square test or Fisher’s exact test when appropriated. Student’s t-test was implemented for normally distributed continuous data and the Mann–Whitney U test for non-normally distributed continuous data. The longitudinal comparison over time was performed using AUC analysis and multivariable mixed-effects generalized linear regression models. Kaplan–Meier curves were generated to assess the cumulative incidence of normalization of parameters over time; hazard ratios (HRs) were calculated, and comparisons were performed using the log–rank test. Finally, the evolution of a categorical variable over time (e.g., different symptom severity levels) employed multivariable mixed-effects generalized regression models for ordinal data. Missing data was managed using the last observation carried forward (LOCF) imputation method. The significance threshold (α) was set at 0.05.

## 3. Results

### 3.1. Population

A total of 60 patients were enrolled, 30 in the Intervention Group and 30 in Control Group. The baseline values of the features analyzed in the study are summarized in Table 1. The mean age of the population was 48.51 (44.66–52.38) years. The population consisted of 46 (76.67%) women and 14 (23.33%) men, with an F-to-M ratio of approximately 3:1. About one-third of the patients (21 (35%)) were active smokers. As per protocol, all patients had a baseline thyroid profile consistent with overt hyperthyroidism, with inhibited TSH levels in 100% of cases (0.007 (0.005–0.009) mUI/L), a mean fT4 of 26.82 (23.30–30.33) ng/L, and a mean fT3 of 9.39 (6.20–19.20) ng/L. All patients showed positive TRAb levels at baseline, with a mean TRAb titer of 11.89 (8.94–14.84) UI/L. No statistically significant difference was observed between the two study groups at baseline regarding demographic and anthropometric variables, negative prognostic factors for GD, history of conditions with symptoms overlapping thyrotoxicosis, or baseline thyroid profile.

### 3.2. Follow–up, Resolution, and Definitive Therapies

Twenty–five patients (41.67%) experienced spontaneous resolution at a mean follow–up time of 11.30 (9.73–12.88) months, whereas 13 cases (21.67%) required referral for definitive therapy at a mean time of 15.92 (12.00–19.84) months. Specifically, 4 patients (6.67%) were referred to radioactive iodine therapy (RAI), and 9 patients (15%) underwent thyroidectomy (Table 2).

A significantly higher rate of patients experienced spontaneous resolution in the Intervention Group during active follow–up (19 (63.33%) vs. 4 (13.33%), OR = 11.22 (3.35–46.11), *p* < 0.001). No difference was observed in terms of time to resolution or indication to definitive therapy (Table 2).

No negative prognostic factor, including male gender, smoking habit, baseline TRAb, fT3 and fT4 levels, was significantly associated with spontaneous resolution (Supplementary Table S1). On the other hand, the implementation of a generalized logistic regression model, proved the Intervention Group to be the only factor associated with spontaneous resolution (β = 3.01, *p* < 0.001), even when evaluated together with the main prognostic factors (Table 3).

### 3.3. Thyroid-Stimulating Hormone (TSH)

A longitudinal analysis of TSH revealed an increasing trend over time in both the Control Group and Intervention Group. At baseline, all patients exhibited suppressed TSH levels (*C:* 0.006 (0.004–0.009) vs. *I:* 0.008 (0.007–0.010) mUI/L, *p* = 0.184), which increased to median levels of 1.619 (0.727–2.511) mUI/L and 1.796 (0.768–2.823) mUI/L (*p* = 0.7910) at 24 months for the Control and Intervention Groups, respectively.

The trend of TSH during treatment did not differ between groups, as the comparison of the median areas under the curve (mAUC) derived from the time-course curves (Supplementary Figure S1) did not reveal any significant difference (*p* = 0.900).

The event “normalization” of TSH was defined as the achievement of TSH levels above the LLN for the reference laboratory (TSH > 0.35 mUI/L). Overall, stable TSH normalization occurred in 63.33% of patients, with a median time to event of 12.00 (10.00–14.00) months. The log-rank test did not demonstrate differences among the two curves (*p* = 0.654) (Supplementary Figure S2).

### 3.4. Free Thyroid Hormones (fT3 and fT4)

A time-dependent reduction in thyroid function indices (fT3 and fT4) was observed in both the Control and Intervention Groups. By the second follow-up at 4 months, mean values had returned to the normal range in both groups (*C_fT_*_3_*:* 4.02 (3.20–4.85) vs. *I_fT_*_3_: 3.81 (2.88–4.74) ng/L; *C_fT_*_4_: 11.00 (8.36–13.66) vs. *C_fT_*_4_: 11.33 (8.78–13.89) ng/L).

These findings were further supported by the analysis of the trends with mAUC, which showed no statistically significant difference between groups (p_fT3_ = 0.147 and p_fT4_ = 0.132) (Supplementary Figures S3a and S4a).

The normalization of fT3 and fT4 was later defined based on the achievement of hormone levels below the laboratory’s ULN (fT3 < 3.9 ng/L; fT4 < 14.8 ng/L). Cumulative incidence curves for each event were constructed (Supplementary Figures S3b and S4b). However, the log-rank test did not reveal statistically significant differences in either case (p_fT3_ = 0.722; p_fT4_ = 0.778).

### 3.5. TSH Receptor Antibodies (TRAb)

At baseline, all patients had TRAb levels above the ULN, with no significant difference between groups (*C:* 12.15 (8.71–15.59) vs. *I*: 11.18 (6.37–15.99) UI/L, *p* = 0.924). A progressive decrease in TRAb levels was observed over time in both groups (at 24 months, *C*: 8.40 (4.73–12.07) vs. *I*: 7.32 (2.47–12.17) UI/L).

**Figure 1:**
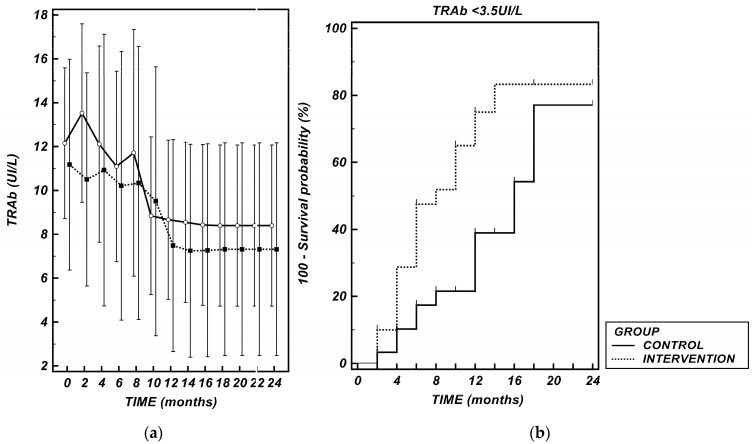
**TRAb**: (**a**) Serum TRAb (UI/L) levels over time in the two study groups. Data are presented as mean ± 95% CI. The comparison of median AUCs derived from the time-course curves did not reveal any significant difference between the groups (*p* = 0.079); (**b**) Kaplan–Meier curves showing the time to normalization of TRAb levels (TRAb < 3.5 UI/L) in the two study groups. The log-rank test did not demonstrate differences among the two curves (*p* = 0.016). TRAb: thyrotropin-receptor antibodies.

The analysis of the mAUC revealed no statistically significant difference between groups (*p* = 0.079) (Figure 1a).

TRAb negativity was defined as the achievement of TRAb levels below the laboratory’s ULN (TRAb < 3.5 UI/L). Survival analysis showed that the Intervention Group achieved negativity significantly faster (HR 2.35 (1.14–4.81), *p* = 0.016). Mean time-to-event revealed 13.55 (11.32–15.78) months for the Control Group and 9.10 (7.04–11.17) months for the Intervention Group (Figure 1b).

A multivariable mixed-effects generalized linear regression model was implemented, with TRAb levels as the dependent variable, and study group, treatment duration, methimazole (MMI) dosage, and their interactions as independent variables. The model output is shown in Table 4. After accounting for individual random variability, the following independent variables were found to be statistically significant:

- MMI dosage, as an independent variable (β = −0.270, *p* < 0.001);
- Treatment duration, as an independent variable (β = −0.231, *p* < 0.001);
- The interaction between Intervention Group and MMI dosage, (β = −0.246, *p* = 0.005).

Other interaction terms were not statistically significant.

### 3.6. Methimazole (MMI) Dosage

All patients initiated therapy according to protocol; therefore, baseline dosage was 0 mg for all participants. The median MMI dose increased at the first follow-up visit at 2 months (*C*: 11.33 (9.14–13.52) mg vs. *I*: 11.08 (8.38–13.79) mg, *p* = 0.884), followed by a gradual reduction over time.

**Figure 2:**
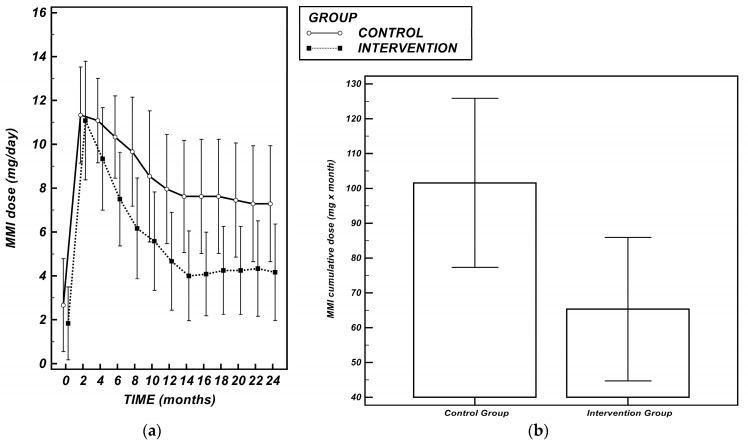
**MMI**: (**a**) MMI daily dose (mg/day) over time in the two study groups. Data are presented as mean ± 95% CI. The comparison of median AUCs derived from the time-course curves revealed a significantly lower dose in the Intervention Group over time (*p* = 0.014). MMI: methimazole; (**b**) Bar plots represent the mean cumulative MMI dose (expressed in mg × months) in the control and intervention groups, with 95% confidence intervals. The cumulative MMI dose was calculated as the sum of monthly daily MMI dosages across the follow-up period, according to a method analogous to the pack-years approach used for cumulative tobacco exposure. The intervention group required a significantly lower cumulative MMI dose compared to the control group (*p* = 0.023).

When assessing the mean cumulative MMI dose required throughout the entire follow–up period, it was significantly lower in the Intervention Group (*C*: 101.58 (77.30–125.86) mg × month vs. *I*: 65.33 (44.72–85.94) mg × month, *p* = 0.023) (Figure 2b).

The Intervention Group confirmed a stably lower trend of MMI dose over time based on the analysis of the mAUC (*p* = 0.014) (Figure 2a).

### 3.7. Symptoms and Quality of Life: Symptom Score (SS)

At baseline, no significant difference in overall Symptom Score (SS) was observed between the study groups (*C:* 14.37 (12.45–16.31) points vs. *I*: 14.50 (12.20–16.80) points, *p* = 0.928). Over time, both groups showed a progressive reduction in symptom burden.

**Figure 3:**
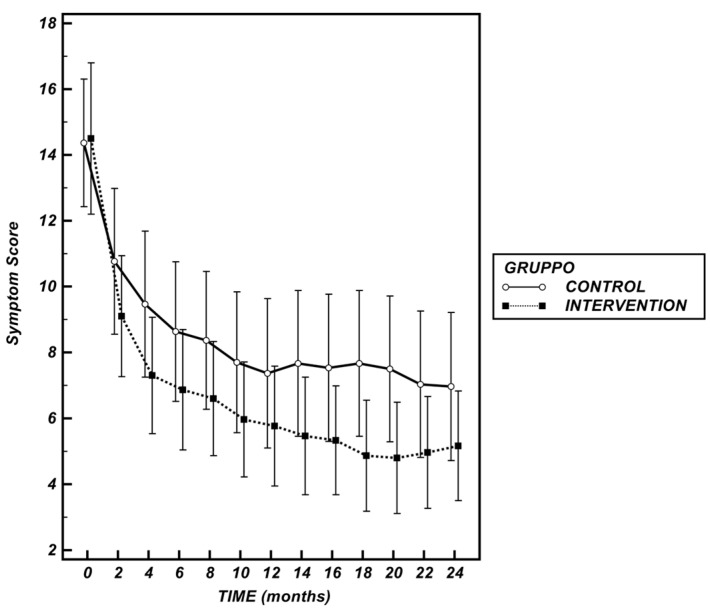
**Symptom Score (points) over time in the two study groups.** Data are presented as mean ± 95% CI. The comparison of median AUCs derived from the time-course curves revealed a higher cumulative symptomatic burden in the Control Group compared to the Intervention Group; however, this difference did not reach statistical significance (*p* = 0.249).

The analysis of the mAUC revealed a higher cumulative symptomatic burden trend in the Control Group compared to Intervention Group; however, this difference was not significant (*p* = 0.249) (Figure 3).

This was later confirmed at a multivariable mixed-effects generalized linear regression model, with SS as the dependent variable, and study group, treatment duration, MMI dosage, and their interactions as independent variables. The Intervention Group did not exert any significant dependent or independent effect (Supplementary Table S2).

### 3.8. Symptoms and Quality of Life: Individual Items

To evaluate the specific impact of the treatment in individual symptoms reported by patients at various follow–up time points, a series of generalized mixed–effects ordinal regression models was applied. In each model, the dependent variable was the severity level of the reported symptom, while the independent variables included treatment duration/follow–up time, MMI dosage, study group, and interactions.

The analyses revealed that the intervention was associated with a significant reduction in the severity of specific hyperthyroid-related symptoms, particularly trembling, irritability, mood lability, heat intolerance, and exertional dyspnea, while no measurable benefit was observed for others such as palpitations, anxiety, insomnia, excessive sweating, or impaired concentration.

Specifically, the following were noted:

- For *irritability*, the intervention proved both an independent effect (*p* < 0.001) on reducing the symptom and a synergistic effect with MMI dosage and time (MMI dose × Group [I]: *p* < 0.001; Time × Group [I]: *p* < 0.001; MMI dose × Time × Group [I]: *p* = 0.006) (Supplementary Table S4);
- For *tremor*, *mood lability,* and *heat intolerance*, the effect was only dependent on time (Time × Group [I]: *p* = 0.009, *p* < 0.001, *p* = 0.024, respectively) (Supplementary Tables S5, S6 and S9);
- For *exertional dyspnea*, again, the effect was dependent on both time and MMI dosage (Time × Group [I]: *p* = 0.006; MMI dose × Time × Group [I]: *p* < 0.001) (Supplementary Table S11).

No role was revealed for palpitations, anxiety, insomnia, excessive sweating, and impaired concentration (Supplementary Tables S3, S7, S8, S10 and S12).

## 4. Discussion

The aim of our study was to evaluate, in a randomized controlled design, the effects of adding a combined supplement containing LCT and Se to standard therapy with MMI in patients with overt GD. To the best of our knowledge, this is the first and only study conducted in this specific clinical setting. Indeed, while several studies have investigated the use of supplements containing either LCT or Se alone, only one trial has assessed the use of the combined supplement. However, that study focused on a population with subclinical GD and did not examine the supplement as an adjunct to standard MMI therapy [38].

In our population, 41.67% of patients achieved spontaneous remission, whereas 21.67% required definitive treatment. These findings are in line with previously reported data in European populations [1]. The median time to spontaneous remission was 11.30 (9.73–12.88) months, which is consistent with the literature. Conversely, the median time to definitive treatment was 15.92 (12.00–19.84) months. Such a finding, likely reflects clinicians’ adherence to international guidelines, which recommend evaluating the need for definitive treatment between 12 and 18 months of therapy [1].

The first noteworthy result was the spontaneous remission rate, which was significantly higher in the Intervention Group (19 (63.33%) vs. 4 (13.33%), *p* < 0.001), even after adjusting for major negative prognostic factors (male sex, smoking status, and baseline TRAb, fT3, and fT4 levels).

The Intervention Group showed a higher remission rate (63.33%) compared to data reported in the literature (40–60%) [1], while the Control Group observed a particularly low remission rate (13.33%), which falls below commonly reported values [1]. This discrepancy may reflect the relatively small sample size. As remission rate was not a primary endpoint, the study was likely underpowered to detect differences for this specific outcome. Baseline characteristics were comparable between groups, and we believe that all major factors were adequately accounted for; nonetheless, the presence of unmeasured confounders cannot be entirely ruled out.

Studies evaluating the outcome of spontaneous remission rates in the context of supplementation are surprisingly scarce. Only two studies evaluating Se supplementation reported data on remission rates, with contrasting results. Wang et al. observed higher remission rates in patients with recurrent GD treated with Se (52.3% vs. 25.0%) [36], whereas Kahaly et al. reported nearly equivalent remission rates between Se and placebo groups (41% vs. 45%) [30]. High serum concentrations of Se and selenoprotein P were observed following Se supplementation and allowed a temporal analysis of its effects on individual selenium status. However, increases in serum Se were not correlated with treatment response or relapse rates. Notably, both studies involved relatively small sample sizes and short intervention periods. The absence of reported remission rates or of significant differences between groups may in part be attributed to the short follow-up durations, which did not exceed 12 months and may therefore have captured only partial or unconfirmed remission events [39].

None of the trials assessing the isolated use of LCT or the combination of LCT and Se have investigated this parameter [15,38].

It was therefore essential to further investigate the various effects of the combined supplement on the disease.

In our study, no significant differences were observed between the two treatment groups in terms of thyroid function indices (TSH, fT3, fT4), as well as in their median trends over time and in normalization rates. The lack of impact on thyroid function is consistent with the nuclear antagonistic properties of L-carnitine (LCT). The only available study evaluating the combined LCT and Se supplement [38], conducted in a population with subclinical GD conveyed comparable results. Similar findings have been reported in studies assessing isolated LCT supplementation in humans, all of which were conducted against placebo and in the absence of MMI, both in earlier research and in the only recent trial [13,14,15].

Evidence on isolated Se supplementation, however, remains inconsistent. In a prospective, randomized, double-blind, placebo-controlled trial conducted in Sweden (a Se-deficient region), Calissendorff et al. reported improved biochemical control of thyroid dysfunction, with reductions in fT4 at 18 and 36 weeks and an increase in TSH at 18 weeks [32]. Consistent findings were observed by Wang et al. in a prospective study from China, where fT4 and fT3 levels were lower in the Se-treated group than in controls after two months of therapy [36].

Conversely, two recent interventional studies showed opposite results. Kahaly et al. conducted a similar trial in Germany, a Se-sufficient region, reporting reduced thyroid hormone concentrations, albeit without statistically significant differences [30]. These findings align with negative results from an Italian Se-sufficient cohort, where Se supplementation during the first three months of therapy showed no impact on clinical or hormonal parameters [24].

These observations suggest that selenium sufficiency may play a key role in modulating the effect of its supplementation on thyroid hormone profiles, as has also been noted in Graves’ orbitopathy (GO) [40].

Recently, Zheng et al. conducted a meta-analysis on the subject, combining data from several available studies. The meta-analysis found a beneficial effect of Se in the biochemical control of hyperthyroidism: patients treated with MMI plus Se supplementation achieved euthyroidism more rapidly than controls. A statistically significant reduction in fT3 and fT4 levels was observed at three and six months in the Se groups, but not at nine months. Additionally, Se supplementation was consistently associated with a significant increase in TSH at six months, but not at three or nine months [35].

In our study, the most notable effect was observed on TRAb levels. Although the Intervention Group did not exhibit significantly lower TRAb concentrations during follow-up, we found a significant synergistic effect between the supplement and MMI dose. This translated into consistently lower MMI dosages over time and a markedly reduced cumulative dose throughout the entire follow-up period.

This finding was further supported by a two-fold higher likelihood of earlier TRAb negativity in the Intervention Group. Specifically, survival analysis revealed a median time to TRAb negativity of 9.10 (7.04–11.17) months in the Intervention Group, which was shorter than the classic incidence peak described around 12 months [1].

Studies and meta-analyses investigating the effect of Se supplementation on TRAb levels consistently report a reduction in antibody levels, in agreement with our findings, although with varying treatment durations and dosages [34,35,36].

This aspect cannot be directly compared with the study by Nordio et al., as those authors did not evaluate TRAb levels. However, they reported a significant reduction in anti-thyroglobulin and anti-thyroid peroxidase antibody levels [38], in line with previous data on selenium supplementation in chronic autoimmune thyroiditis [33].

This evidence, combined with our findings, may shed new light on a potential synergistic effect of combined Se and LCT supplementation, suggesting an additional immunomodulatory role for LCT, beyond its known peripheral antagonism of thyroid hormone action.

Regarding symptom burden and quality of life (QoL), our findings did not reveal a significant improvement in overall symptomatology, for which both treatment duration and methimazole dosage appeared to play predominant roles. However, the supplement showed a significant impact on specific symptoms by enhancing the effect of time and/or MMI dose, including: tremor, irritability, mood lability, heat intolerance, and exertional dyspnea. No significant effects were observed for palpitations, anxiety, insomnia, excessive sweating, or impaired concentration.

In this regard, our findings partially diverge from the available literature. Both the study by Nordio et al. and studies evaluating LCT supplementation alone have reported significant reduction in global and item-specific symptom scores [15,38]. However, all these studies lacked comparison with standard therapy, which undoubtedly plays a central role in symptom control. Therefore, it is noteworthy that our study demonstrated a reduction in a subset of symptoms independent of standard treatment, although it remains evident that MMI and/or beta-blockers are irreplaceable in managing clinical symptoms in these patients.

By contrast, studies evaluating the effect of selenium alone, in addition to standard MMI therapy, did not show any significant improvement in symptom burden, despite evidence of a biochemical effect on thyroid hormone levels [24,32].

These findings further support the added value of the combination therapy, in which the peripheral antagonism of thyroid hormone action provided by L-carnitine appears to contribute, beyond the standard inhibitory effect of MMI, to a partial alleviation of several hyperthyroidism-related symptoms.

The major strength of our study lies in its novelty. To date, the only study evaluating the combined supplement was conducted in patients with subclinical GD and did not include standard MMI therapy as a comparator, while other trials on LCT alone lacked comparison with MMI. The literature on Se supplementation is more extensive but remains heterogeneous in its conclusions.

### Limitations of Our Study

- The absence of a validated QoL questionnaire is one limitation, as it may have exposed the results to several potential biases, including measurement bias due to reduced psychometric robustness and increased susceptibility to interpretation variability by respondents. Furthermore, the absence of prior validation may reduce the comparability of our findings with those obtained in other settings using standardized tools. Nevertheless, the choice of a non-validated questionnaire allowed us to design questions specifically tailored to the clinical features and symptomatic spectrum of hyperthyroidism. At the main follow-up time points, the questionnaire demonstrated good internal consistency (Supplementary Materials—Internal Consistency of QoL Questionnaire (Cronbach’s alpha)), supporting its reliability within the context of our study. In addition, since the same instrument was employed in the study that primarily inspired our protocol, its use facilitated a direct and meaningful comparison with the available literature.
- The lack of placebo and blinded control group is another limitation that could have exposed the study to expectation bias, especially impacting subjective outcomes on symptoms and quality of life. However, it is precisely in these domains that our results have already proven to be of limited significance, thereby minimizing the potential impact of this limitation on the overall study conclusions.
- The absence of baseline and longitudinal serum measurements of LCT and selenium levels is another limitation. These measurements are frequently included in similar studies to correlate clinical outcomes with effective biochemical availability of the supplements.

## 5. Conclusions

In conclusion, our findings provide evidence supporting the clinical benefit of adding combined LCT and Se supplementation to MMI in the treatment of overt GD. We observed a faster decline in TRAb levels, a higher rate of biochemical remission, and the need for substantially lower MMI doses. The role of LCT as a peripheral antagonist of thyroid hormones seems limited compared with that of MMI, contributing to the reduction of only some of the evaluated symptoms without significant differences in biochemical thyroid profiles. Taken together, these effects may translate into meaningful clinical advantages, including shorter disease duration, lower cumulative antithyroid drug exposure, better symptom control, and earlier TRAb normality, that could positively influence TRAb-related prognostic outcomes, such as the need for second-line therapies, responsiveness to radioiodine treatment, and the course of Graves’ orbitopathy.

## Figures and Tables

**Table 1:** **Baseline values for each feature of both the population as a whole and separate groups.** Categorical variables are expressed as n (%), normally distributed continuous variables as mean (95% CI), and non-normally distributed continuous variables as median (interquartile range). Group comparisons were performed using Fisher’s exact test for categorical variables with fewer than five observations, the chi-square test for categorical variables with more than five observations, Student’s t-test for normally distributed continuous variables (marked with * if log-transformed), and the Mann–Whitney U test for non-normally distributed continuous variables. Statistical significance was set at α = 0.05. TSH: thyrotropin, fT3: free thyronine, fT4: free thyroxine, TRAb: thyrotropin-receptor antibodies.

	Population (n= 60)	Control Group (n = 30)	Intervention Group (n = 30)	*p*
**Age (years)**	48.51 (44.66–52.38)	48.03 (41.95–51.12)	49.00 (43.87–54.13)	0.804
**Gender (female)**	46 (76.67%)	23 (76.67%)	23 (76.67%)	0.760
**Body Mass Index**	23.15 (22.21–24.14)	23.47 (20.36–25.00)	22.84 (21.56–24.20)	0.517
**Smoking habit**				0.585
*Active smokers*	21 (35%)	9 (25%)	12 (30%)	0.588
*Never smoked*	32 (53.33%)	18 (60%)	14 (50%)	0.438
*Stopped smoking*	7 (11.67%)	3 (15%)	4 (20%)	1.000
TSH * (mUI/L)	0.008 (0.008–0.010)	0.006 (0.004–0.009)	0.008 (0.007–0.010)	0.184
fT3 * (ng/L)	9.39 (6.20–19.20)	12.10 (6.12–20.00)	8.80 (6.15–14.43)	0.282
**fT4 (ng/L)**	26.82 (23.30–30.33)	29.03 (23.53–34.52)	24.53 (19.99–29.07)	0.203
**TRAb (UI/L)**	11.89 (8.94–14.84)	12.15 (8.71–15.79)	11.87 (6.80–16.93)	0.748

**Table 2:** **Records from the follow-up for features and events.** Categorical variables are expressed as n (%), normally-distributed continuous variables as mean (95% CI), and non-normally-distributed continuous variables as median (interquartile range). Group comparisons were performed using Fisher’s exact test for categorical variables with fewer than 5 observations, the Chi-square test for categorical variables with more than 5 observations, Student’s t-test for normally distributed continuous variables (marked with if log-transformed), and the Mann–Whitney U test for non-normally distributed continuous variables. Statistical significance was set at α = 0.05.

	Population (n = 60)	Control Group (n = 30)	Intervention Group (n = 30)	*p*
**Spontaneous resolution**	25 (41.67%)	4 (13.33%)	19 (63.33%)	**<0.001**
**Time to spontaneous** **resolution**	11.30 (9.73–12.88)	11.50 (7.50–15.51)	11.26 (9.38–13.14)	0.909
**Definitive treatment**	13 (21.67%)	7 (23.33%)	6 (20%)	0.185
**Type of definitive** **treatment**				0.559
*Radioiodine*	4 (6.67%)	3 (10%)	1 (3.33%)	0.606
*Thyroidectomy*	9 (15%)	4 (13.33%)	5 (16.67%)	0.709
**Time to definitive** **treatment**	15.92 (12.00–19.84)	16.29 (11.46–21.11)	15.50 (6.85–24.57)	0.838

**Table 4:** **Generalized mixed-effects linear regression model assessing the longitudinal trend of TRAb levels.** The model includes study group, follow-up duration (months), and daily methimazole dose (mg/day) as fixed effects, along with their interaction terms. A random intercept was included for each patient (ID). The analysis shows that longer treatment duration (*p* < 0.001), higher MMI dose (*p* < 0.001), and the interaction between intervention group and MMI dose (*p* = 0.005) are all significantly associated with lower TRAb levels over time. No significant effect was observed for the study group alone or other interaction terms. α = 0.04. S.E.: standard error, TRAb: thyrotropin-receptor antibodies, MMI: methimazole.

Generalized Linear Mixed Model Fit by Maximum Likelihood (Laplace Approximation)				
Formula: TRAb ~ GROUP × TIME. × MMI.dose. + (1|ID)				
Family: Gaussian (Identity);				
Fixed Effects	Estimate	S.E.	t-Value	*p*-Value
GROUP [INTERVENTION]	0.882	3.034	0.291	0.771
**TIME.**	−0.231	0.043	−5.321	**<0.001**
**MMI.dose.**	−0.270	0.062	4.364	**<0.001**
GROUP [INTERVENTION]:TIME.	0.037	0.057	0.648	0.517
**GROUP [INTERVENTION]:MMI.dose.**	−0.246	0.087	−2.836	**0.005**
TIME.:MMI.dose.	0.005	0.005	1.028	0.304
GROUP [INTERVENTION]:TIME.:MMI.dose	−0.004	0.007	−0.514	0.607

**Table 3:** **Generalized logistic regression model for predicting spontaneous resolution**. Age, intervention group, male gender, smoking habit, and fT4 and TRAb levels at baseline were evaluated as independent variables. Only the Intervention Group proved to positively predict the probability of spontaneous resolution (*p* < 0.001). α = 0.05. S.E.: standard error, fT4: free thyroxine, TRAb: thyrotropin-receptor antibodies.

Generalized Linear Model				
Formula: RESOLUTION ~ AGE + FT4 + SMOKE + SEX + TRAB + GROUP.				
Family = binomial(logit)				
**Overall Model Fit**				
Null model Log Likelihood 0.025; Full model Log Likelihood 51.257				
Chi-squared 23.767; Degrees of Freedom 6; **Significance level *p* = 0.0006**				
	**Estimate**	**S.E.**	**z-Value**	***p*-Value**
AGE	−0.047	0.030	−1.544	0.123
FT4	−0.004	0.029	−0.146	0.884
SMOKE [Yes]	−1.312	0.825	−1.590	0.112
SEX [Male]	1.152	0.881	1.308	0.191
TRAB	−0.031	0.038	−0.807	0.420
**GROUP [INTERVENTION]**	3.015	0.824	3.658	**<0.001**

## Data Availability

The original contributions presented in this study are included in the article/Supplementary Materials. Further inquiries can be directed to the corresponding author(s).

## Supplementary Materials

The following supporting information can be downloaded at: https://www.mdpi.com/article/10.3390/nu17162693/s1, Figure S1. Serum TSH (mIU/L) levels over time in the two study groups. Data are presented as mean ± 95% CI. The comparison of median AUCs derived from the time-course curves did not reveal any significant difference between the groups (*p* = 0.900); Figure S2. Kaplan–Meier curves showing the time to normalization of TSH levels (TSH > 0.35 mIU/L) in the two study groups. The log-rank test did not demonstrate differences among the two curves (*p* = 0.654); Figure S3. (a) K Serum fT3 (ng/L) levels over time in the two study groups. Data are presented as mean ± 95% CI. The comparison of median AUCs derived from the time-course curves did not reveal any significant difference between the groups (*p* = 0.147); (b) Kaplan–Meier curves showing the time to normalization of fT3 levels (fT3 < 3.9 ng/L) in the two study groups. The log-rank test did not demonstrate differences among the two curves (*p* = 0.722); Figure S4. (a) Serum fT4 (ng/L) levels over time in the two study groups. Data are presented as mean ± 95% CI. The comparison of median AUCs derived from the time-course curves did not reveal any significant difference between the groups (*p* = 0.132); (b). Kaplan–Meier curves showing the time to normalization of fT4 levels (fT4 < 14.8 ng/L) in the two study groups. The log-rank test did not demonstrate differences among the two curves (*p* = 0.778); Table S1. Distribution of variables known to be negative prognostic factors in relation to the achievement of spontaneous resolution during follow-up. Categorical variables are expressed as n (%), while continuous variables are presented as median (interquartile range). Group comparisons were performed using Fisher’s exact test for categorical variables and the Mann–Whitney U test for continuous variables. α = 0.05; Table S2. Generalized mixed-effects linear regression model assessing the longitudinal trend of Symptom Score (SS). The model includes study group, follow-up duration (months), and daily methimazole dose (mg/day) as fixed effects, along with their interaction terms. A random intercept was included for each patient (ID). The analysis shows that longer treatment duration (*p* < 0.001), higher MMI dose (*p* < 0.001), and the interaction between treatment duration and MMI dose (*p* = 0.003) are all significantly associated with lower SS. No significant effect was observed for the study group alone or other interaction terms. α = 0.05; Table S3. Ordinal mixed model (cumulative link model) examining the impact of treatment group, time, and MMI dose on the severity of the item “Palpitations”. While MMI dose and time showed significant main effects (*p* < 0.001), along with their interaction, no significant effect was observed for the intervention group or its interactions. The model includes random intercepts for individual patients (ID). α = 0.05; Table S4. Ordinal mixed model (cumulative link model) examining the impact of treatment group, time, and MMI dose on the severity of the item “Irritability”. All the variables and their interactions showed significant main effects (*p* < 0.001), except the interaction between time and MMI dose. The model includes random intercepts for individual patients (ID). α = 0.05.; Table S5. Ordinal mixed model (cumulative link model) examining the impact of treatment group, time, and MMI dose on the severity of the item “Tremor”. Time and MMI dose showed significant independent effect (*p* < 0.001). Group [I] showed significant effect only over time (*p* = 0.009). The model includes random intercepts for individual patients (ID). α = 0.05; Table S6. Ordinal mixed model (cumulative link model) examining the impact of treatment group, time, and MMI dose on the severity of the item “Mood lability”. Time and the interaction of time and intervention group showed significant effect (*p* < 0.001). The model includes random intercepts for individual patients (ID). α = 0.05; Table S7. Ordinal mixed model (cumulative link model) examining the impact of treatment group, time, and MMI dose on the severity of the item “Anxiety”. While MMI dose and time showed significant main effects (*p* < 0.001, *p* = 0.03), along with their interaction (*p* < 0.001), no significant effect was observed for the intervention group or its interactions. The model includes random intercepts for individual patients (ID). α = 0.05; Table S8. Ordinal mixed model (cumulative link model) examining the impact of treatment group, time, and MMI dose on the severity of the item “Excessive sweating”. While MMI dose and time showed significant main effects (*p* = 0.044, *p* < 0.001), along with their interaction (*p* = 0.025), no significant effect was observed for the intervention group or its interactions. The model includes random intercepts for individual patients (ID). α = 0.05; Table S9. Ordinal mixed model (cumulative link model) examining the impact of treatment group, time, and MMI dose on the severity of the item “Heat intolerance”. Time and the interaction of time and intervention group showed significant effect (*p* < 0.001; *p* = 0.024). The model includes random intercepts for individual patients (ID). α = 0.05; Table S10. Ordinal mixed model (cumulative link model) examining the impact of treatment group, time, and MMI dose on the severity of the item “Insomnia”. Only time as an independent factor showed significant effect (*p* < 0.001). The model includes random intercepts for individual patients (ID). α = 0.05; Table S11. Ordinal mixed model (cumulative link model) examining the impact of treatment group, time, and MMI dose on the severity of the item “Exertion dyspnea”. All the variables and their interactions showed significant main effects (*p* < 0.001), except the intervention group as an independent factor and its interaction with MMI dose. The model includes random intercepts for individual patients (ID). α = 0.05; Table S12. Ordinal mixed model (cumulative link model) examining the impact of treatment group, time, and MMI dose on the severity of the item “Impaired concentration”. No variable showed significant effects. Only time approached significance, without reaching it (*p* = 0.0823). The model includes random intercepts for individual patients (ID). α = 0.05; Table S13. Reliability analysis of the questionnaire at baseline (Cronbach’s alpha). The table reports the internal consistency of the scale, indicating good reliability (Cronbach’s alpha = 0.79–0.80). Average inter-item correlations are moderate (Avg R = 0.28), suggesting that items are correlated but not redundant. The signal-to-noise ratio (S/N = 3.9) indicates that the scale predominantly measures the intended construct. The standard error of alpha (ASE = 0.04) shows a precise estimate. The 95% confidence intervals for alpha, calculated using Feldt and Duhachek methods, confirm acceptable reliability in all scenarios (Feldt CI: 0.71–0.86; Duhachek CI: 0.72–0.87); Table S14. Reliability analysis of the questionnaire at 6 months (Cronbach’s alpha). The table shows excellent internal consistency for the scale, with Cronbach’s alpha values of 0.96–0.98. Average inter-item correlations are high (Avg R = 0.73), indicating strong coherence among items. The signal-to-noise ratio (S/N = 26) demonstrates a very clear measurement of the intended construct. The standard error of alpha (ASE = 0.007) confirms a highly precise estimate. The 95% confidence intervals for alpha, computed using Feldt and Duhachek methods, further support the robustness of the scale (Feldt CI: 0.94–0.97; Duhachek CI: 0.95–0.97); Table S15. Reliability analysis of the questionnaire at 12 months (Cronbach’s alpha). The analysis shows good internal consistency, with Cronbach’s alpha values of 0.89–0.91. Average inter-item correlation is moderate-to-high (Avg R = 0.45), suggesting a coherent but not redundant set of items. The signal-to-noise ratio (S/N = 8.2) indicates a solid capacity to measure the intended construct. The standard error of alpha (ASE = 0.022) points to a precise estimate. The 95% confidence intervals, calculated with both Feldt and Duhachek methods, confirm the reliability of the scale (Feldt CI: 0.84–0.93; Duhachek CI: 0.85–0.93).
