# Supplementary material for: Adding L-Carnitine and Selenium to Methimazole in Graves’ Disease: A Prospective Randomized Trial on Thyroid Markers and Quality of Life
Source: Nutrients. 2025 Aug 20;17(16):2693. doi: 10.3390/nu17162693 (PMC12389566; doi:10.3390/nu17162693)
Supplement: Supplementary file 1 [file nutrients-17-02693-s001.zip › nutrients-3814636-supplementary.pdf]

Supplementary Materials

Table S1. Distribution of variables known to be negative prognostic factors in relation to the achievement of spontaneous resolution during follow-up. Categorical variables are expressed as n (%), while continuous variables are presented as median (interquartile range). Group comparisons were performed using Fisher's exact test for categorical variables and the Mann–Whitney U test for continuous variables. $\alpha = 0.05$.

	**Spontaneous resolution (n = 23)**	**No resolution (n = 37)**	***p.***
**Age (years)**	47.43 (40.24 - 54.53)	49.19 (44.52 - 53.85)	0.662
**Gender (M)**	7 (30.43%)	7 (18.92%)	0.477
**Smoking (Active)**	6 (26.09%)	15 (40.54%)	0.388
**TRAb**	9.17 (5.63 – 12.71)	13.55 (9.28 – 17.82)	0.113
**fT3 (ng/L)**	11.80 (8.88 – 14.73)	11.43 (9.30 – 13.56)	0.830
**fT4 (ng/L)**	25.61 (19.67 – 31.54)	27.54 (22.97 – 32.10)	0.599

Table S2. Generalized mixed-effects linear regression model assessing the longitudinal trend of Symptom Score (SS). The model includes study group, follow-up duration (months), and daily methimazole dose (mg/day) as fixed effects, along with their interaction terms. A random intercept was included for each patient (ID). The analysis shows that longer treatment duration ($p < 0.001$), higher MMI dose ($p < 0.001$), and the interaction between treatment duration and MMI dose ($p = 0.003$) are all significantly associated with lower SS. No significant effect was observed for the study group alone or other interaction terms. $\alpha = 0.05$.

Generalized linear mixed model fit by maximum likelihood (Laplace Approximation)
Formula: SS ~ MMI.dose. * TIME. * GROUP + (1 | ID)
Family: gaussian (identity);

**Fixed effects**	**Estimate**	**Std. Error**	**t-value**	**p-value**	
**MMI.dose.**	-0,193	0,042	-4,645	**<0,001**	***
**TIME.**	-0,587	0,058	-10,087	**<0,001**	***
GROUP[T.INTERVENTION]	-1,911	1,875	-1,019	0,308	
**MMI.dose.:TIME.**	0,018	0,006	2,978	**0,003**	**
MMI.dose.:GROUP[T.INTERVENTION]	0,016	0,058	0,273	0,785	
TIME.:GROUP[T.INTERVENTION]	-0,077	0,077	-1,001	0,317	
MMI.dose.:TIME.:GROUP[T.INTERVENTION]	0,003	0,009	0,323	0,47	

Table S3. Ordinal mixed model (cumulative link model) examining the impact of treatment group, time, and MMI dose on the severity of the item "Palpitations". While MMI dose and time showed significant main effects ($p < 0.001$), along with their interaction, no significant effect was observed for the intervention group or its interactions. The model includes random intercepts for individual patients (ID). $\alpha = 0.05$

Cumulative Link Mixed Model fitted with the Laplace Approximation
Formula: PALPITATIONS ~ MMI.dose. * TIME * GROUP + (1 | ID)

**Fixed effects**	**Estimate**	**Std. Error**	**t-value**	**p-value**	
**MMI.dose.**	-0,185	0,039	-4,734	**<0,001**	***
**TIME.**	-0,424	0,058	-7,330	**<0,001**	***
GROUP[T.INTERVENTION]	-1,064	0,946	-1,124	0,261	
**MMI.dose.:TIME.**	0,019	0,005	3,664	**<0,001**	***

MMI.dose.:GROUP[T.INTERVENTION]	0,029	0,054	0,538	0,590	
TIME.:GROUP[T.INTERVENTION]	-0,099	0,078	-1,278	0,201	
MMI.dose.:TIME.:GROUP[T.INTERVENTION]	0,008	0,008	0,998	0,318	
**Threshold coefficients:**					

	Estimate	Std. Error	z-value
0∣1	-3,658	0,704	-5,197
1∣2	0,038	0,684	0,055
2∣3	3,175	0,705	4,501

Table S4. Ordinal mixed model (cumulative link model) examining the impact of treatment group, time, and MMI dose on the severity of the item "Irritability". All the variables and their interactions showed significant main effects ($p < 0.001$), except the interaction between time and MMI dose. The model includes random intercepts for individual patients (ID). $\alpha = 0.05$.

Cumulative Link Mixed Model fitted with the Laplace Approximation
Formula: IRRITABILITY ~ MMI.dose. * TIME * GROUP + (1 ∣ ID)

Fixed effects	Estimate	Std. Error	t-value	p-value	
**MMI.dose.**	-0,095	0,005	-18,780	**<0,001**	***
**TIME.**	-0,214	0,005	-41,493	**<0,001**	***
**GROUP[T.INTERVENTION]**	-1,039	0,005	-192,555	**<0,001**	***
MMI.dose.:TIME.	-0,001	0,003	-0,297	0,767	
**MMI.dose.:GROUP[T.INTERVENTION]**	-0,028	0,005	-5,366	**<0,001**	***
**TIME.:GROUP[T.INTERVENTION]**	-0,145	0,005	-28,026	**<0,001**	***
**MMI.dose.:TIME.:GROUP[T.INTERVENTION]**	0,010	0,004	2779,000	**0,005**	**

Threshold coefficients:

	Estimate	Std. Error	z-value
0∣1	-4,358	0,238	-18,330
1∣2	-0,094	0,005	-17,510
2∣3	4,741	0,005	899,280

Table S5. Ordinal mixed model (cumulative link model) examining the impact of treatment group, time, and MMI dose on the severity of the item "Tremor". Time and MMI dose showed significant independent effect ($p < 0.001$). Group [I] showed significant effect only over time ($p = 0.009$). The model includes random intercepts for individual patients (ID). $\alpha = 0.05$.

Cumulative Link Mixed Model fitted with the Laplace Approximation
Formula: TREMOR ~ MMI.dose. * TIME * GROUP + (1 ∣ ID)

Fixed effects	Estimate	Std. Error	t-value	p-value	
**MMI.dose.**	-0,141	0,038	-3,741	**<0,001**	***
**TIME.**	-0,261	0,057	-4,578	**<0,001**	***
GROUP[T.INTERVENTION]	-0,961	0,848	-1,133	0,257	
MMI.dose.:TIME.	0,000	0,007	-0,026	0,980	
MMI.dose.:GROUP[T.INTERVENTION]	0,076	0,055	1,385	0,166	
**TIME.:GROUP[T.INTERVENTION]**	-0,216	0,083	-2,606	**0,009**	**
MMI.dose.:TIME.:GROUP[T.INTERVENTION]	0,012	0,010	1,155	0,248	

Threshold coefficients:

	Estimate	Std. Error	z-value
0∣1	-2,100	0,612	-3,430
1∣2	1,642	0,606	2,711

2∣3	3,880	0,656	5,916

Table S6. Ordinal mixed model (cumulative link model) examining the impact of treatment group, time, and MMI dose on the severity of the item "Mood lability". Time and the interaction of time and intervention group showed significant effect (p < 0.001). The model includes random intercepts for individual patients (ID). α = 0.05.

Cumulative Link Mixed Model fitted with the Laplace Approximation

Formula: MOOD LABILITY ~ MMI.dose. * TIME * GROUP + (1 ∣ ID)

Fixed effects	Estimate	Std. Error	t-value	p-value	
**TIME.**	-0,192	0,055	-3,496	**<0,001**	***
GROUP[T.INTERVENTION]	-1,025	1,259	-0,814	0,416	
MMI.dose.:TIME.	0,006	0,006	1,126	0,260	
MMI.dose.:GROUP[T.INTERVENTION]	0,016	0,055	0,289	0,772	
**TIME.:GROUP[T.INTERVENTION]**	-0,311	0,081	-3,846	**<0,001**	***
MMI.dose.:TIME.:GROUP[T.INTERVENTION]	0,005	0,010	0,543	0,587	
**TIME.**	-0,192	0,055	-3,496	**<0,001**	***

Threshold coefficients:

	Estimate	Std. Error	z-value
0∣1	-3,654	0,924	-3,953
1∣2	0,675	0,910	0,742
2∣3	8,047	1,124	7,157

Table S7. Ordinal mixed model (cumulative link model) examining the impact of treatment group, time, and MMI dose on the severity of the item "Anxiety". While MMI dose and time showed significant main effects (p < 0.001, p = 0.03), along with their interaction (p < 0.001), no significant effect was observed for the intervention group or its interactions. The model includes random intercepts for individual patients (ID). α = 0.05.

Cumulative Link Mixed Model fitted with the Laplace Approximation

Formula: ANXIETY ~ MMI.dose. * TIME * GROUP + (1 ∣ ID)

Fixed effects	Estimate	Std. Error	t-value	p-value	
**MMI.dose.**	-0,082	0,038	**-2,136**	**0,033**	*
**TIME.**	-0,321	0,056	**-5,690**	**<0,001**	***
GROUP[T.INTERVENTION]	-0,993	1,270	-0,782	0,435	
**MMI.dose.:TIME.**	0,023	0,006	**3,897**	**<0,001**	***
MMI.dose.:GROUP[T.INTERVENTION]	-0,010	0,055	-0,189	0,850	
TIME.:GROUP[T.INTERVENTION]	-0,002	0,074	-0,022	0,983	
MMI.dose.:TIME.:GROUP[T.INTERVENTION]	-0,012	0,008	-1,401	0,161	

Threshold coefficients:

	Estimate	Std. Error	z-value
0∣1	-4,184	0,937	-4,464
1∣2	0,122	0,918	0,133
2∣3	6,060	0,990	6,123

Table S8. Ordinal mixed model (cumulative link model) examining the impact of treatment group, time, and MMI dose on the severity of the item "Excessive sweating". While MMI dose and time showed significant main effects (p = 0.044, p < 0.001), along with their interaction (p = 0.025), no significant effect was observed for the intervention group or its interactions. The model includes random intercepts for individual patients (ID). α = 0.05.

**Cumulative Link Mixed Model fitted with the Laplace Approximation**					
Formula: SWEAT ~ MMI.dose. * TIME * GROUP + (1 ∣ ID)					
**Fixed effects**	**Estimate**	**Std. Error**	**t-value**	**p-value**	
**MMI.dose.**	-0,074	0,037	**-2,010**	**0,044**	*
**TIME.**	-0,334	0,056	**-5,957**	**<0,001**	***
GROUP[T.INTERVENTION]	-0,440	1,013	-0,435	0,664	
**MMI.dose.:TIME.**	0,012	0,005	**2,233**	**0,026**	*
MMI.dose.:GROUP[T.INTERVENTION]	0,006	0,051	0,119	0,905	
TIME.:GROUP[T.INTERVENTION]	0,047	0,073	0,654	0,513	
MMI.dose.:TIME.:GROUP[T.INTERVENTION]	0,006	0,008	0,724	0,469	

Threshold coefficients:

	**Estimate**	**Std. Error**	**z-value**
0∣1	-2,763	0,744	-3,713
1∣2	0,374	0,737	0,508
2∣3	4,200	0,762	5,509

Table S9. Ordinal mixed model (cumulative link model) examining the impact of treatment group, time, and MMI dose on the severity of the item "Heat intolerance". Time and the interaction of time and intervention group showed significant effect ($p < 0.001$; $p = 0.024$). The model includes random intercepts for individual patients (ID). $\alpha = 0.05$.

**Cumulative Link Mixed Model fitted with the Laplace Approximation**					
Formula: HEAT ~ MMI.dose. * TIME * GROUP + (1 ∣ ID)					
**Fixed effects**	**Estimate**	**Std. Error**	**t-value**	**p-value**	
MMI.dose.	0,005	0,036	0,147	0,883	
**TIME.**	-0,174	0,052	**-3,328**	**0,001**	***
GROUP[T.INTERVENTION]	0,505	0,996	0,507	0,612	
MMI.dose.:TIME.	-0,001	0,005	-0,274	0,784	
MMI.dose.:GROUP[T.INTERVENTION]	-0,055	0,050	-1,099	0,272	
**TIME.:GROUP[T.INTERVENTION]**	-0,159	0,071	**-2,257**	**0,024**	*
MMI.dose.:TIME.:GROUP[T.INTERVENTION]	0,012	0,008	1,542	0,123	

Threshold coefficients:

	**Estimate**	**Std. Error**	**z-value**
0∣1	-1,909	0,729	-2,619
1∣2	1,075	0,726	1,480
2∣3	4,737	0,766	6,186

Table S10. Ordinal mixed model (cumulative link model) examining the impact of treatment group, time, and MMI dose on the severity of the item "Insomnia". Only time ad an independent factor showed significant effect ($p < 0.001$). The model includes random intercepts for individual patients (ID). $\alpha = 0.05$.

**Cumulative Link Mixed Model fitted with the Laplace Approximation**					
Formula: INSOMNIA ~ MMI.dose. * TIME * GROUP + (1 ∣ ID)					
**Fixed effects**	**Estimate**	**Std. Error**	**t-value**	**p-value**	
MMI.dose.	-0,010	0,036	-0,266	0,790	
**TIME.**	-0,232	0,054	**-4,320**	**<0,001**	***
GROUP[T.INTERVENTION]	-0,263	1,094	-0,240	0,810	
MMI.dose.:TIME.	0,003	0,005	0,619	0,536	
MMI.dose.:GROUP[T.INTERVENTION]	-0,048	0,054	-0,905	0,365	

TIME.:GROUP[T.INTERVENTION]	0,048	0,072	0,665	0,506	
MMI.dose.:TIME.:GROUP[T.INTERVENTION]	0,001	0,008	0,081	0,935	
**Threshold coefficients:**					
	**Estimate**	**Std. Error**	**z-value**		
0|1	-0,876	0,789	-1,110		
1|2	2,027	0,795	2,551		
2|3	5,155	0,824	6,259		

Table S11. Ordinal mixed model (cumulative link model) examining the impact of treatment group, time, and MMI dose on the severity of the item "Exertion dyspnea". All the variables and their interactions showed significant main effects ($p < 0.001$), except the intervention group as an independent factor and its interaction with MMI dose. The model includes random intercepts for individual patients (ID). $\alpha = 0.05$.

**Cumulative Link Mixed Model fitted with the Laplace Approximation**					
Formula: DYSPNEA ~ MMI.dose. * TIME * GROUP + (1 ∣ ID)					
**Fixed effects**	**Estimate**	**Std. Error**	**t-value**	**p-value**	
**MMI.dose.**	-0,055	0,003	-18,004	**<0,001**	***
**TIME.**	-0,584	0,003	-190,517	**<0,001**	***
GROUP[T.INTERVENTION]	-0,842	0,632	-1,332	0,183	
**MMI.dose.:TIME.**	0,030	0,002	19,011	**<0,001**	***
MMI.dose.:GROUP[T.INTERVENTION]	-0,015	0,039	-0,369	0,712	
**TIME.:GROUP[T.INTERVENTION]**	0,225	0,057	3,919	**<0,001**	***
**MMI.dose.:TIME.:GROUP[T.INTERVENTION]**	-0,027	0,008	-3,379	**0,001**	***
**Threshold coefficients:**					
	**Estimate**	**Std. Error**	**z-value**		
0|1	-1,584	0,003	-470,100		
1|2	2,648	0,003	854,200		
2|3	7,162	0,003	2.381,100		

Table S12. Ordinal mixed model (cumulative link model) examining the impact of treatment group, time, and MMI dose on the severity of the item "Impaired concentration". No variable showed significant effects. Only time approached significance, without reaching it ($p = 0.0823$). The model includes random intercepts for individual patients (ID). $\alpha = 0.05$.

**Cumulative Link Mixed Model fitted with the Laplace Approximation**					
Formula: CONCENTRATION ~ MMI.dose. * TIME * GROUP + (1 ∣ ID)					
**Fixed effects**	**Estimate**	**Std. Error**	**t-value**	**p-value**	
MMI.dose.	-0,018	0,044	-0,400	0,689	
TIME.	-0,221	0,128	-1,736	0,083	.
GROUP[T.INTERVENTION]	-0,233	0,812	-0,287	0,774	
MMI.dose.:TIME.	-0,007	0,014	-0,473	0,636	
MMI.dose.:GROUP[T.INTERVENTION]	-0,075	0,064	-1,176	0,240	
TIME.:GROUP[T.INTERVENTION]	-0,198	0,170	-1,164	0,244	
MMI.dose.:TIME.:GROUP[T.INTERVENTION]	0,012	0,020	0,603	0,546	
**Threshold coefficients:**					
	**Estimate**	**Std. Error**	**z-value**		
0|1	-1,673	0,607	-2,758		
1|2	1,381	0,600	2,301		
2|3	4,135	0,733	5,640		

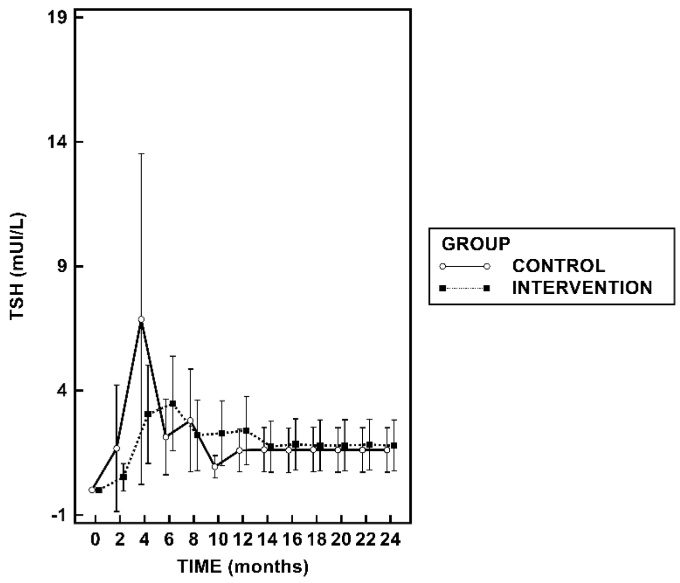

Figure S1. Serum TSH (mIU/L) levels over time in the two study groups. Data are presented as mean ± 95% CI. The comparison of median AUCs derived from the time-course curves did not reveal any significant difference between the groups (p = 0.900).

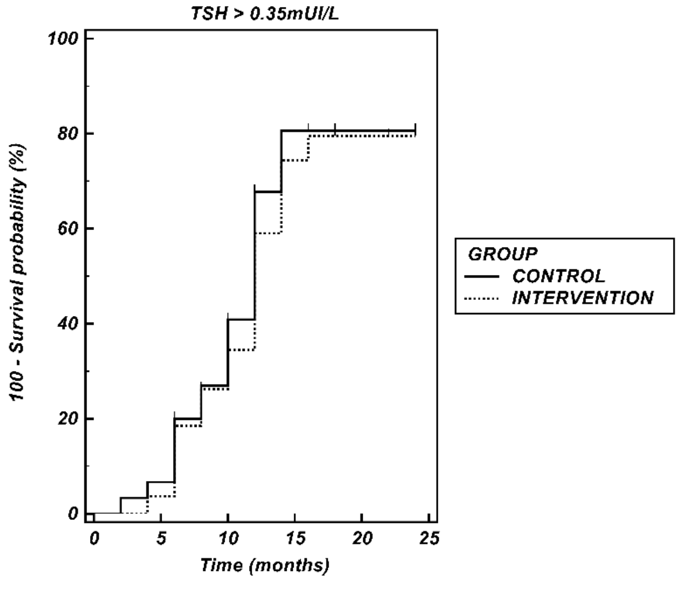

Figure S2. Kaplan–Meier curves showing the time to normalization of TSH levels (TSH > 0.35 mIU/L) in the two study groups. The log-rank test did not demonstrate differences among the two curves (p = 0.654).

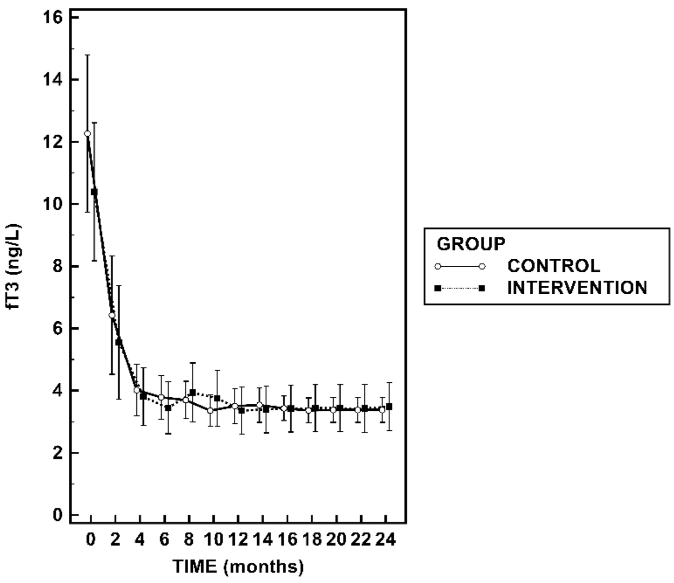

Figure S3a. K Serum fT3 (ng/L) levels over time in the two study groups. Data are presented as mean ± 95% CI. The comparison of median AUCs derived from the time-course curves did not reveal any significant difference between the groups (p = 0.147).

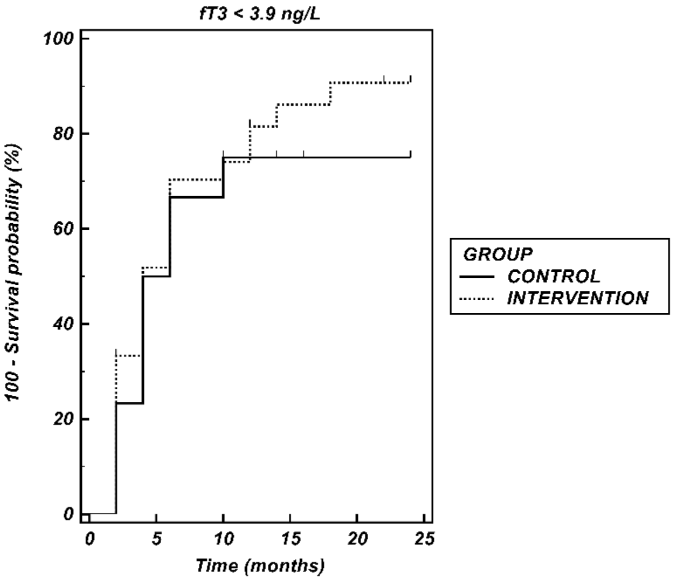

Figure S3b. Kaplan–Meier curves showing the time to normalization of fT3 levels (fT3 < 3.9 ng/L) in the two study groups. The log-rank test did not demonstrate differences among the two curves (p = 0.722).

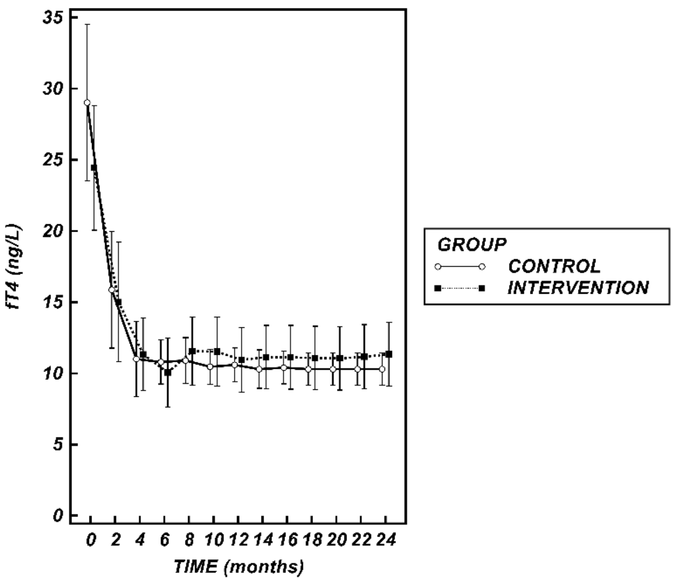

Figure S4a. Serum fT4 (ng/L) levels over time in the two study groups. Data are presented as mean ± 95% CI. The comparison of median AUCs derived from the time-course curves did not reveal any significant difference between the groups (p = 0.132).

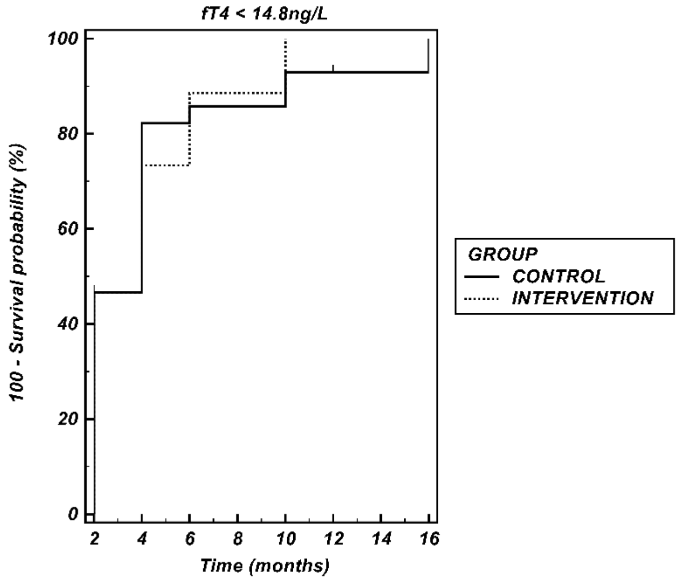

Figure S4b. Kaplan–Meier curves showing the time to normalization of fT4 levels (fT4 < 14.8 ng/L) in the two study groups. The log-rank test did not demonstrate differences among the two curves (p = 0.778).

Internal Consistency of QoL Questionnaire (Cronbach's alpha)

The questionnaire we implemented in our study has not undergone validation process and was specifically designed for the study, based on the most common and debilitating symptoms of hyperthyroidism, which often drive the clinician to use additional drugs. The same tool was employed in the study that primarily inspired our protocol [38], and its use facilitated a direct and meaningful comparison with the available literature. Therefore, we assessed its internal consistency using Cronbach's alpha on three main follow-up times (baseline, 6 months, and 12 months) in order to ensure the reliability of the instrument, to verify the coherence of responses across items and to support the interpretability of the derived scores within our study population.

Tables C1, C2, and C3 summarize the results of this analysis, which, in our population, demonstrated a precise and reliable, yet not redundant, estimate for each follow-up time.

Table S13. Reliability analysis of the questionnaire at baseline (Cronbach's alpha). The table reports the internal consistency of the scale, indicating good reliability (Cronbach's alpha = 0.79–0.80). Average inter-item correlations are moderate (Avg R = 0.28), suggesting that items are correlated but not redundant. The signal-to-noise ratio (S/N = 3.9) indicates that the scale predominantly measures the intended construct. The standard error of alpha (ASE = 0.04) shows a precise estimate. The 95% confidence intervals for alpha, calculated using Feldt and Duhachek methods, confirm acceptable reliability in all scenarios (Feldt CI: 0.71–0.86; Duhachek CI: 0.72–0.87).

**Reliability analysis (Cronbach's alpha) – Time 0**								
**Raw alpha**	**Std. alpha**	**G6(smc)**	**Avg R**	**S/N**	**ASE**	**Mean**	**SD**	**Median R**
0.79	0.8	0.83	0.28	3.9	0.04	2.2	0.6	0.27
**95% CI**	**Lower**	**Alpha**	**Upper**					
Feldt	0.71	0.79	0.86					
Duhachek	0.72	0.79	0.87					

Table S14. Reliability analysis of the questionnaire at 6 months (Cronbach's alpha). The table shows excellent internal consistency for the scale, with Cronbach's alpha values of 0.96–0.98. Average inter-item correlations are high (Avg R = 0.73), indicating strong coherence among items. The signal-to-noise ratio (S/N = 26) demonstrates a very clear measurement of the intended construct. The standard error of alpha (ASE = 0.007) confirms a highly precise estimate. The 95% confidence intervals for alpha, computed using Feldt and Duhachek methods, further support the robustness of the scale (Feldt CI: 0.94–0.97; Duhachek CI: 0.95–0.97).

**Reliability analysis (Cronbach's alpha) – Time 6**								
**Raw alpha**	**Std. alpha**	**G6(smc)**	**Avg R**	**S/N**	**ASE**	**Mean**	**SD**	**Median R**
0.96	0.96	0.98	0.73	26	0.007	3.2	0.82	0.73
**95% CI**	**Lower**	**Alpha**	**Upper**					
Feldt	0.94	0.96	0.97					
Duhachek	0.95	0.96	0.97					

Table S15. Reliability analysis of the questionnaire at 12 months (Cronbach's alpha). The analysis shows good internal consistency, with Cronbach's alpha values of 0.89–0.91. Average inter-item correlation is moderate-to-high (Avg R = 0.45), suggesting a coherent but not redundant set of items. The signal-to-noise ratio (S/N = 8.2) indicates a solid capacity to measure the intended construct. The standard error of alpha (ASE = 0.022) points to a precise estimate. The 95% confidence intervals, calculated with both Feldt and Duhachek methods, confirm the reliability of the scale (Feldt CI: 0.84–0.93; Duhachek CI: 0.85–0.93).

**Reliability analysis (Cronbach's alpha) – Time 12**

Raw alpha	Std. alpha	G6(smc)	Avg R	S/N	ASE	Mean	SD	Median R
0.89	0.89	0.91	0.45	8.2	0.022	3.4	0.56	0.46

95% CI	Lower	Alpha	Upper
Feldt	0.84	0.89	0.93
Duhachek	0.85	0.89	0.93
